# Measuring the impact of an acute visiting scheme on emergency department attendances – a pre-post cohort design

**DOI:** 10.1186/s12913-021-06557-3

**Published:** 2021-05-28

**Authors:** Axel Kaehne, Paula Keating

**Affiliations:** 1 Reader Health Services Research, Medical School, Edge Hill University, Ormskirk, L39 4QP UK; 2 Head of Women’s and Children’s Health Care, Faculty of Health, Social Care and Medicine, Edge Hill University, Ormskirk, UK

**Keywords:** Primary care, ED, Hospital admissions, Emergency admissions, Acute visiting scheme

## Abstract

**Background:**

Emergency department (ED) attendances are contributing to rising costs of the National Health Service (NHS) in England. Critically assessing the impact of new services to reduce emergency department use can be difficult as new services may create additional access points, unlocking latent demand. The study evaluated an Acute Visiting Scheme (AVS) in a primary care context. We asked if AVS reduces overall ED demand and whether or not it changed utilisation patterns for frequent attenders.

**Method:**

The study used a pre post single cohort design. The impact of AVS on all-cause ED attendances was hypothesised as a substitution effect, where AVS duty doctor visits would replace emergency department visits. Primary outcome was frequency of ED attendances. End points were reduction of frequency of service use and increase of intervals between attendances by frequent attenders.

**Results:**

ED attendances for AVS users rose by 47.6%. If AVS use was included, there was a more than fourfold increase of total service utilisation, amounting to 438.3%. It shows that AVS unlocked significant latent demand. However, there was some reduction in the frequency of ED attendances for some patients and an increase in time intervals between ED attendances for others.

**Conclusion:**

The study demonstrates that careful analysis of patient utilisation can detect a differential impact of AVS on the use of ED. As the new service created additional access points for patients and hence introduces an element of choice, the new service is likely to unlock latent demand. This study illustrates that AVS may be most useful if targeted at specific patient groups who are most likely to benefit from the new service.

## Background

Emergency department (ED) use is rising in England contributing to resource constraints of the National Health Service (NHS) [1–3]. There is substantial discussion in the literature about the causes of a rise in emergency attendances and how to reduce it [3–16]. Since 2013, primary care organisations in England, so-called Clinical Commissioning Groups (CCG), are responsible for a significant part of the NHS budget, including payments for ED attendances. As commissioners and budget holders in the NHS, CCGs have a strong interest to implement changes that may reduce the number and cost of ED attendances. There is, however, a lack of evidence on what works for whom with the exception of studies focusing on chronic disease patient groups [2, 8, 17]. Acute visiting schemes (AVS) for primary care patients are becoming an increasingly popular tool to address ED attendances. AVS are commonly ‘within hours’ services, with a GP practice led triage and referral system and duty doctors conducting home visits to patients to prevent unscheduled visits to ED.

The first pilot of an AVS was run by a GP practice in the North West of England in 2009 and received considerable media attention. Whilst there was no independent audit of service outcomes or independent evaluation of the pilot, the media reported a 30% drop in emergency admissions.^1^

The debate about how to reduce emergency department use is plagued by lack of terminological clarity. The terms ‘avoidable ED attendance’ and ‘preventable admissions’ are often used interchangeably. Regarding ED attendances, researchers and commissioners often speak of ‘avoidable’, ‘preventable’ or ‘unnecessary’ ED attendances without defining the terms further [18]. An additional problem relates to the aims and objectives of newly commissioned services, such as AVS. It is not always clear what the role of AVS within the primary care service configuration is. Since GPs already conduct home visits, AVS may replace or duplicate an already existing service. In addition, it may be questioned whether AVS are supposed to reduce ‘avoidable ED attendances’ or ‘avoidable admissions to emergency departments’. This blurring of function, purpose and scope of AVS makes it difficult to assess its effectiveness.

There is also limited knowledge about patient behaviour in preference sensitive service environments where patients have choice of service. There is some evidence that waiting times and timely access to GPs affect the decision making of patients visiting ED departments [4, 11]. Yet, patient decisions may also be influenced by factors such as availability of transport, proximity to GP practices or acute treatment centres, or patients’ perceived urgency of care [10].

There is some evidence that clinical indicators such as poly-pharmacy predict repeated service use [19] and that significant geographical variations exist [20]. However, whilst administrative data allows identifying long term trends [21] there is little research on all-cause ED attendances due to the multifactorial modelling required to demonstrate efficacy of interventions for this type of service use.

The aim of this study was to identify the impact of AVS on all-cause ED attendances, including attendances leading to hospital admissions. The study hypothesized that AVS would lead to a reduction of all types of emergency department attendances amongst the service population. When commissioning an additional service, the hope of commissioners is that a cheaper service will replace a more expensive service of equal or similar care quality. In this paper, we call this a simple substitution effect which conventionally underpins cost savings calculations by commissioners.

Given the above mentioned difficulties in defining and conceptualising potential effects of AVS, the present evaluation conceptualised service impact not only through a simple substitution effect of AVS on all emergency department attendances, but, also as measured by (1) frequency of attendances at ED and (2) the time interval lapsed between ED attendances. Our primary outcomes were number of visits of ED. Secondary outcomes were the time between ED use for frequent attenders. We formulated the following research questions.

1. Do frequent attenders of emergency hospital departments use emergency departments less when using AVS?
2. Does using an AVS increase the time interval between ED visits or reduce the frequency of ED attendances of frequent attenders?

We note that there is some debate about the meaning and utility of the term ‘frequent attender’ [8, 11, 13, 18, 22]. We hope that clearly defining frequent attenders in our context below will remove the terminological ambiguities and address any potential sensitivities.

## Methods

### Study design

The project used a pre-post single cohort design. We obtained administrative data from the local CCG for all ED attendances at all local hospitals in the CCG footprint for a 6 months period from May to October 2014. We also obtained from the CCG the routine AVS service data for its initial 3 months of operation, the period from August to October 2014.

### Setting

The study was conducted in an area serviced by an NHS Clinical Commissioning Group (CCG) in the North West of England. The NHS is a tax funded health service in the UK which is free at the point of use for all citizens and residents of the UK. Health service governance, service commissioning and planning is devolved to the four home nations and diverges significantly across the four constituent parts of the UK, England, Wales, Scotland and Northern Ireland. The commissioning group in our study had a footprint of 37 GP practices at time of evaluation. Duty doctors could refer to three hospitals with emergency departments. The registered patient population of the study site was 197,000. GP practices were recruited by the CCG. There was no additional payment incentive for GPs if they participated. GP practices were self-selecting, and all GP practices in the CCG volunteered to take part in this pilot. There was no information available on the reasons why GPs would take part. All relevant processes and technology were already part of routine GP practices and hence training was not deemed necessary.

### Service Specification

AVS are based on single triage and referral systems with the GP practice acting as gatekeepers. When the GP practice receives a call from a patient they are offered a GP appointment. If the patient indicates that they have urgent care needs the receptionist logs the call for potential referral to AVS or home visit. The GP then assesses the need of the patient and makes a referral to AVS if appropriate. AVS duty doctors are then supposed to conduct a home visit within a specific time, most commonly within 60 to 120 min from time of referral. During the home visit, the duty doctor takes (electronic) notes for follow up medical care, logs the time of the visit and indicates whether the visit has prevented attendance at an ED department. Where necessary, admission to hospital is initiated.

Referrals to the present AVS were supposed to be restricted to patients aged 75 plus, yet exceptions were permitted where urgent care needs required the attention by a duty doctor. The AVS operated parallel to conventional home visits by local GPs.

### Participants

We utilised a single cohort design. The study included all adult patients serviced by the AVS and attending ED within the study period. Although the AVS service was designed for patients aged 75 and older, we applied no exclusions since we noticed that, despite this service specification, doctors attended to adult patients of a much broader age range. Given this specification slippage, we wanted to capture the effect of AVS on all frequent attenders and not only on those aged 75 and older. For analysis, we created a cohort of patients who had received a visit from AVS in the 3 months the services started operating, August to October 2014. All ED attendance records for those patients were then identified for both the previous three and the following 3 months when the AVS was operational, capturing their total service utilisation for 6 months. Using this single cohort design, patients were in effect their own control as they used ED in the first 3 months, and, in the second period of time, could use both services.

### Data Analysis

All data were pseudonymised through the Data Management and Integration Centre (DMIC) which allowed linking data sets through individual patient identifiers. Four AVS incidence records were excluded because they contained implausible or incomplete information. Data were analysed with SPSS (IBM SPSS Statistics for Windows; Version 20.0. Armonk, NY: IBM Corp.). Data endpoints were attendance of ED and use of AVS represented by visits of duty doctors.

Primary outcome was the total number of ED attendance. Secondary outcomes were frequency of attendance for individual patients and time intervals between ED visits.

Given the terminological and conceptual ambiguity, the evaluation did not differentiate between what constitutes ‘avoidable’, ‘preventable’ or ‘unnecessary’ attendances but included all-cause attendances.

Analysis was conducted for two 3 months periods, t_1_ and t_2_. The 3 months period prior to implementation of AVS was t_1_ (May to July 2014) whilst t_2_ comprised the 3 months post-implementation period (August to October 2014). Total number of visits to ED and total number of AVS visits were calculated for all patients in our cohort, and compared between time periods. We hypothesized that AVS visits would reduce ED attendances by virtue of replacing one service with another. We used the Chi Square test to check if any particular patient demographic characteristic predicted choice of service in time t_2_.

In a second step, we hypothesized that the AVS may impact not directly by reducing total numbers of ED utilisation but by reducing the frequency of ED visits or increasing the time lapsed (intervals) between ED visits for frequent attenders. From our first cohort of AVS users, we created a cohort of all frequent attenders in t_1_ including only those patients who had attended ED within t_1_ at least twice. We then compared the service utilisation rates of this cohort of patients with t_2_ to see if the frequency of their visits declined or the intervals between their ED visits increased.

We used a cohort stratification strategy to distinguish between two groups of frequent attenders: those attending at least twice and up to 9 times, and those attending 10 or more times during t_1_. The stratification point was set for statistical convenience. The most time any patient frequented emergency departments within a 2 week period was 18 times.

We used descriptive statistical analysis to ascertain the potential effect of reducing frequency or increasing intervals between frequent visits by those patients in our cohort and used column diagrams to illustrate the results. We calculated the probability with which patients may return to ED and represented those in relevant graphs. We also tested any correlation between use of AVS and frequency of ED visit.

The study was reviewed by the local University Faculty Research Ethics Committee. As the project was deemed to be a service evaluation using pseudonymised administrative data, it was approved by chair’s action in line with HRA guidance at the time.

## Results

### Descriptive analysis

The mean age of patients was 78.9 years (SD = 13.6). Perceived urgency of medical attention as assessed by triaging GPs explains some outliers in this measure. Mean patient waiting time for AVS duty doctor visit was 62.5 min (SD = 48). Just over 90 % (92.1%) of duty doctor visits did not lead to hospital admissions. Where admission to hospital emergency departments was made (*n* = 77), the majority of referrals led to admission to one specific local hospital (*n* = 71), with small numbers of admissions to two other hospitals (*n* = 4 and *n* = 2 respectively).

Our analysis focused on the number of visits of frequent attenders. There were a total of 810 patients using AVS in t_2_. Their total number of AVS visits was 1009. The number of ED attendances by these patients at time t_1_ was *n* = 347 and increased to *n* = 512 at t_2_. ED attendances for AVS users thus rose by 47.6%. Since AVS use represented an additional service on top of the (increased) emergency department use, the total volume of service use by this cohort for both services in t_2_ was ED_t2_ + AVS_t2_ = 1521 visits. This represented a more than fourfold increase of total service utilisation compared to t_1_ by the cohort, coming in at 438.3%.

We used the Chi Square test to check whether age or gender would predict ED attendance. However no significant association was found, with χ(1) = 715.132, *p* = 1.000 for the association of age with attendance, and χ(1) = 12.969, *p* = .793 for the association of gender and attendance.

Testing the effect of AVS on frequency and interval between attendances during t_2_, we found that, for patients attending emergency departments in t_1_, there was a 1.8% chance that patients frequented emergency departments 10 or 18 times, whereas there was a 17.2% chance that they would attend emergency departments only once up to 9 times.

**Fig. 1:**
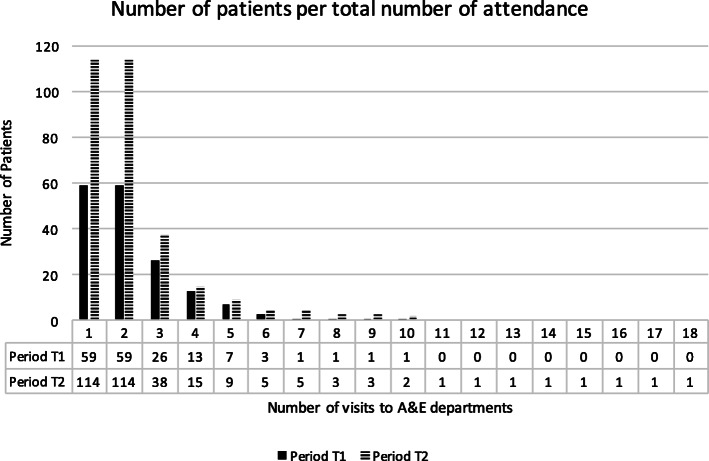
Number of patients per number of attendance

In the second time period (t_2_), patients were less likely to attend emergency departments 10 or more times (1.1%) whereas they were more likely to attend emergency only once or up to 9 times (21.2%). This indicates that there was some movement between the stratified groups of patients with some patients attending less frequently as the AVS started to operate (see frequency table and chart below, Fig. 1). There was however no significant association between frequency of attendance and the use of AVS, with χ(1) = 152.000, *p* = .308.

**Fig. 2:**
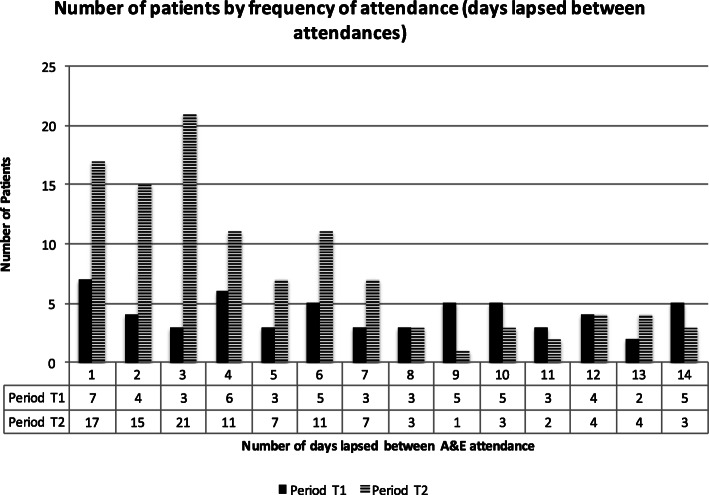
Number of patients by days lapsed between attendances

To provide a more detailed picture of this shift in frequency we examined the hypothesis that AVS would lead to an increase in time intervals between ED attendances for our patient cohort. We conducted a sequential match analysis of ‘frequent attenders’ to identify changes to intervals between ED attendances of patients. The results showed that those patients frequenting emergency departments repeatedly within 9, 10 or 11 days actually attended those departments less frequently in t_2_ compared to t_1_. Other patients showed a significant decrease in intervals between frequent attendances which demonstrated that AVS in fact led to a utilisation increase. We illustrate this in the Fig. 2 below.

## Discussion

Our study demonstrates that simple comparisons of service utilisation rates pre and post implementation of AVS uncovers considerable increases in service utilisation rates. The study thus shows that AVS does not decrease ED utilisation per se. This is echoed in the literature and discussed under the theme of unlocking latent demand. There is considerable evidence that additional primary care access points unlock latent demand and simple substitution effects take a significant period of time to materialise. Consequently, the literature recognises the uncertainties around simple cost trajectories in health care planning [23–25].

However, this does not reveal the whole picture. Investigating our second research question clearly showed that the frequency of attendances decreased for some patients and the intervals between attendances of some frequent attenders increased as well. This varied picture has several implications for pilot programmes such as ours, as well as for evaluating them.

Our study demonstrated that assessing the impact of AVS on ED attendances requires complex modeling of effects. Two factors in particular may require specific attention. First, AVS was an additional service to ED which provided patients with additional options to seek medical care. There is good evidence for the phenomenon of unlocking latent demand in health systems as services increase access to services. Unlocking latent demand is often observed in preference sensitive primary care contexts where additional access points are created and the increase in service utilisation may therefore be entirely expected. Only longitudinal cohort studies may show a tapering off of patient demand. However, the short term nature of many pilots may prevent longitudinal studies. The rapid service commissioning and implementation cycle of transformative health care programmes recently in the NHS, such as the VANGUARD programme, may also mitigate against longitudinal cohort studies [26].

A possible solution may be to employ more sophisticated data mining models with careful patient stratification to identify short term service impact. We tested two potential service effects: a frequency reduction and a frequency interval increase in service use. The analysis showed that there were indeed some effects of the new AVS. There was a considerable impact of the AVS on some patient groups that were frequenting emergency departments repeatedly within a certain number of days, showing an increase in the time intervals between their ED attendances.

Our analysis points to two important areas for future research. Additional services, such as AVS, are unlikely to show quick gains in cost savings or lead to reductions of ED attendances. Longitudinal studies thus need to evidence the length of this initial time period when unlocking latent demand occurs. This should guide policy makers when commissioning pilots of service changes to allow evaluators and researchers to capture long term effects of new services or new interventions [27]. Second, our evaluation demonstrates the need to improve our understanding of patient behaviour and how to influence it in preference sensitive service settings. Service changes may create additional options for patients to access medical care, or improve access to existing services. In universal payer and gatekeeper systems, such as the NHS primary care sector, this introduces an element of choice for patients which requires evaluators to take account of issues of patient behavior and agency when measuring the effect of new services.

Our study has relevance to practice, in particular to commissioning and evaluating primary care services. The analysis showed that a service such as AVS may be effective in the short term if targeted at specific patient populations. The most probable patient groups to show positive impact are high frequency users and in particular those patients who use emergency departments repeatedly within short intervals. Careful monitoring through real time analysis of service use may help commissioners and service planners to target AVS at those patient groups and thus improve the cost-effectiveness of any future AVS.

The study did not investigate patient behaviour, an important driver for service use [28], or participation in the scheme by local GP practices, aspects of service design modeling and implementation that may require more complex evaluative approaches [29]. Since AVS was an optional service in our context, these aspects may influence service utilisation trends significantly, but without any additional data or reliable models of patient behaviour, it would be impossible to quantify these factors [30].

### Limitations

Our study has some limitations. The study was conducted using data drawn from a 6 months period, including data from a 3 months period of a service after imsplementation. Analysis of longitudinal data may improve the robustness of the findings. The study used an observational design. The use of a separate control group would strengthen the quality of evidence in future studies.

The study used a cohort design with stratification by frequency of service use. An alternative approach would have been to use a whole population cohort approach [18]. There are strengths and weaknesses to either approach. The specific aim and objective of the evaluation was to measure the impact of AVS on frequent attenders. A whole population approach would have contained a lot of noise with regard to this evaluation aim. Naturally, the new service served not only frequent attenders but also other patients. Investigating the service use patterns of non-frequent attenders may be useful in a different context.

Another potential weakness of a cohort design is that service use may be episodic. Patients who frequent emergency departments may do this for a particular period of time during which they experience episodes of ill-health. Those episodes may come to pass, which means that the same patients who are frequent attenders for a time may then exhibit different service use patterns. We believe that only a longitudinal study design would have adequately captured the episodic aspect of frequent attenders. Our study data was limited to 6 months period. Within this short period of time, episodic fluctuations to the mean of service use would have been outside the observable data.

However, there may be reasons to challenge the ‘episodic’ nature of frequent attenders in our context. Discussions with clinical staff involved in delivering AVS gave us anecdotal evidence that frequent attenders were unlikely to be episodic users of emergency department but were more likely to be patients with mental health issues or substance addiction.

Last but not least, the types of patients included in the study may exhibit specific service use patterns due to their needs profiles or medical complexities due to age. The study did not analyse the medical needs that led to ED attendance or AVS visits. Going beyond the frequency analysis would have required significant additional resources to code administrative data for each patient. Future research will need to investigate the utility of AVS for patient groups with specific medical and service needs. Targeting AVS in this way may reveal the potential of the service.

## Conclusion

Our study demonstrates that careful modeling and analysis of patient utilisation data can detect a differential impact of AVS on accident and emergency use where additional access points are being created and the new service is unlocking latent demand. Our model may be used to target AVS in the future to specific patient groups who are most likely to benefit from such a service.

## Data Availability

Data sets and output files of the data analysis are available from the authors by request.

## Abbreviations

AVS: Acute visiting scheme
ED: Emergency department
CCG: Clinical commissioning group

## Footnotes

1 http://www.pulsetoday.co.uk/home/practical-commissioning/how-our-acute-visiting-service-reduced-emergency-admissions-by-30-per-cent/20002277.article - .VcSGKIcUZtc (last accessed on 22 August 2018)
